# Untargeted Metabolomics Approach in Halophiles: Understanding the Biodeterioration Process of Building Materials

**DOI:** 10.3389/fmicb.2017.02448

**Published:** 2017-12-11

**Authors:** Justyna Adamiak, Vincent Bonifay, Anna Otlewska, Jan A. Sunner, Iwona B. Beech, Teresa Stryszewska, Stanisław Kańka, Joanna Oracz, Dorota Żyżelewicz, Beata Gutarowska

**Affiliations:** ^1^Institute of Fermentation Technology and Microbiology, Faculty of Biotechnology and Food Sciences, Lodz University of Technology, Lodz, Poland; ^2^Department of Microbiology and Plant Biology, University of Oklahoma, Norman, OK, United States; ^3^Center of Biofilm Engineering, Department of Chemical and Biochemical Engineering, Montana State University, Bozeman, MT, United States; ^4^Institute of Building Materials and Structures, Faculty of Civil Engineering, Cracow University of Technology, Cracow, Poland; ^5^Institute of Food Technology and Analysis, Faculty of Biotechnology and Food Sciences, Lodz University of Technology, Lodz, Poland

**Keywords:** halophilic microorganisms, metabolomics, HPLC/HRMS, CARS microscopy, haloadaptation, biodeterioration, brick

## Abstract

The aim of the study was to explore the halophile metabolome in building materials using untargeted metabolomics which allows for broad metabolome coverage. For this reason, we used high-performance liquid chromatography interfaced to high-resolution mass spectrometry (HPLC/HRMS). As an alternative to standard microscopy techniques, we introduced pioneering Coherent Anti-stokes Raman Scattering Microscopy (CARS) to non-invasively visualize microbial cells. Brick samples saturated with salt solution (KCl, NaCl (two salinity levels), MgSO_4_, Mg(NO_3_)_2_), were inoculated with the mixture of preselected halophilic microorganisms, i.e., bacteria: *Halobacillus styriensis, Halobacillus naozhouensis, Halobacillus hunanensis, Staphylococcus succinus, Marinococcus halophilus, Virgibacillus halodenitryficans*, and yeast: *Sterigmatomyces halophilus* and stored at 28°C and 80% relative humidity for a year. Metabolites were extracted directly from the brick samples and measured via HPLC/HRMS in both positive and negative ion modes. Overall, untargeted metabolomics allowed for discovering the interactions of halophilic microorganisms with buildings materials which together with CARS microscopy enabled us to elucidate the biodeterioration process caused by halophiles. We observed that halophile metabolome was differently affected by different salt solutions. Furthermore, we found indications for haloadaptive strategies and degradation of brick samples due to microbial pigment production as a salt stress response. Finally, we detected changes in lipid content related to changes in the structure of phospholipid bilayers and membrane fluidity.

## Introduction

The durability of building materials, including those of historical value, is influenced by numerous physical, chemical, and biological factors. Among them, salt crystallization, which forms as a result of salt migration with capillary water and subsequent drying out and precipitation, poses a serious threat to the materials' structure (Stryszewska, 2014). Moreover, salt-attacked monuments constitute a suitable habitat for halophilic microorganisms (Piñar et al., 2014; Adamiak et al., 2015; Otlewska et al., 2017) as these can easily adapt to salty micro-niches available on a building's surface, thus contributing to its structural destabilization (Saiz-Jimenez and Laiz, 2000; Piñar et al., 2009; Adamiak et al., 2015, 2016).

Microbial metabolomics has proved to be an emerging field for microorganism identification, mutant screening, and functional gene research, metabolic pathway identification, and microbial engineering. Over the last two decades it has provided new insights on the activities of microbes in response to environmental conditions (Xu et al., 2014; Villas-Boas, 2016). Recently, several outstanding papers on microbial metabolomics have been published (Rochfort, 2005; Mashego et al., 2007; van der Werf et al., 2007; Tang, 2011; Baidoo et al., 2012; Xu et al., 2014; Barkal et al., 2015). To our knowledge, there are only a limited number of reports on biodeterioration and biocorrosion processes (Lenhart et al., 2014; Brauer et al., 2015; Gutarowska et al., 2015; Bonifay et al., 2017; Szulc et al., 2017), and none of them has focused on halophilic microorganisms.

Biodeterioration of building materials due to active growth of microorganisms is another area where microbial metabolomics has much to offer. For example, Gutarowska et al. (2015) used ultra-high performance liquid chromatography coupled to high-resolution mass spectrometry (UPLC/HRMS) to investigate activated pathways of microbial communities inhabiting wood and brick collected from historical objects. Szulc et al. (2017) compared the metabolite profiles of molds on carton-gypsum board using a high-resolution surface-assisted laser desorption/ionization time-of-flight mass spectrometry based on a gold nanoparticle-enhanced target (AuNPET SALDI-ToF-MS) imaging method. Growing awareness of biodeterioration of building materials and halophiles contributing to this phenomenon (Rölleke et al., 1998; Heyrman et al., 1999; Laiz et al., 2000, 2009; Piñar et al., 2001, 2014; Ripka et al., 2006; Ettenauer et al., 2014) has highlighted gaps in our understanding of the metabolic pathways that characterize halophilic communities colonizing historical buildings. At present, there are no studies providing an in-depth metabolomic assessment of halophiles-induced biodeterioration.

A well-known phenomenon found on wall surfaces is the formation of salt efflorescence, which mimics the extreme salty conditions favoring the proliferation of halophiles (Sterflinger and Piñar, 2013). The ability of halophilic microorganisms to cope with osmotic stress in hypersaline environments is an important adaptation. Two different strategies for coping with osmotic stress have been reported. One involves the accumulation of inorganic ions (mostly K^+^ and Cl^−^ rather than Na^+^) and is known as the “salt-in-cytoplasm” strategy. The other, known as the “compatible solute” strategy employs the uptake or synthesis of organic molecules, namely sugars and polyols or α- and β-amino acids with their derivatives (Madigan and Oren, 1999; Xiang et al., 2008; Averhoff and Müller, 2010). When studying the interactions of halophilic microorganisms with building materials, we should take into consideration not only their ability to survive in a high-salt environment (haloadaptation), but also their contribution to the degradation of building materials. A wide range of carotenoid pigments, such as β-carotene, α-bacterioruberin and derivatives, and salinixanthin is produced by halophilic microorganisms (Oren, 2002). They are the major cause of typical rosy stains, significantly influencing the visual appearance of building material (Sterflinger and Piñar, 2013). Organic acids also play an important role, because they contribute to cracking and detachment of building materials (Gutarowska and Czyzowska, 2009). Halophilic microorganisms isolated from salterns, brines, sediments, and soil were shown to produce organic acids; e.g., gluconic acid, 2-ketogluconic, citric acid, formic acid, succinic acid, fumaric acid, and acetic acid (Tomlinson and Hochstein, 1972; Vreeland et al., 1998; Yadav et al., 2015); however, this ability was not confirmed for isolates from a building environment.

When elucidating the biodeterioration caused by halophilic microorganisms, it can be helpful to utilize microscopic methods. However, rapid detection of microbes in their natural environment (Hong et al., 2016) is still a challenge. Here, we report the use of coherent anti-Stokes Raman scattering (CARS) for *in situ* detection of a single living cell. This state-of-the-art technology offers a non-invasive, label-free, and direct way to image biological samples with chemical sensitivity (Cheng et al., 2002; Cheng and Xie, 2004; Rodriguez et al., 2006). It relies on the abundance of CH_2_ groups in lipids and the distinctive CH_2_ stretch vibration frequency at 2,840 cm^−1^ (Folick et al., 2011), thus enabling detection of a single bacterium. CARS requires the input of two laser pulses, a pump-probe beam and a Stokes beam, which interact with the specimen to generate an anti-Stokes field. When the frequency difference between the pump and the Stokes pulses matches the frequency of a certain molecular vibration, a strong signal is generated, allowing molecule-specific imaging (Zhang et al., 2015; Hong et al., 2016). Visualizing lipid structures in live cells may therefore represent an alternative to standard procedures employed in biodeterioration studies.

The present work focused on untargeted metabolomics using high-performance liquid chromatography coupled to high-resolution mass spectrometry (HPLC/HRMS) to explore the halophile metabolome. Mass spectrometry is known to be the most sensitive and selective technique among many platforms. This work demonstrates that a combination of what might be termed “community metabolomics” and state-of-the-art CARS microscopy has an enormous potential for the comprehensive characterization of biodeterioration on salt-attacked buildings. Therefore, the aim of the research was to: (1) identify groups of metabolites produced by mixed cultures of halophilic microorganisms that may be potentially responsible for degradation of brick; (2) reveal the pattern of metabolic responses of halophilic microorganisms to severe saline conditions; and (3) visualize live cells of halophilic microorganisms with CARS.

## Experimental procedures

### Brick samples preparation

#### Exposure to salty corrosive environment

**Figure 1:**
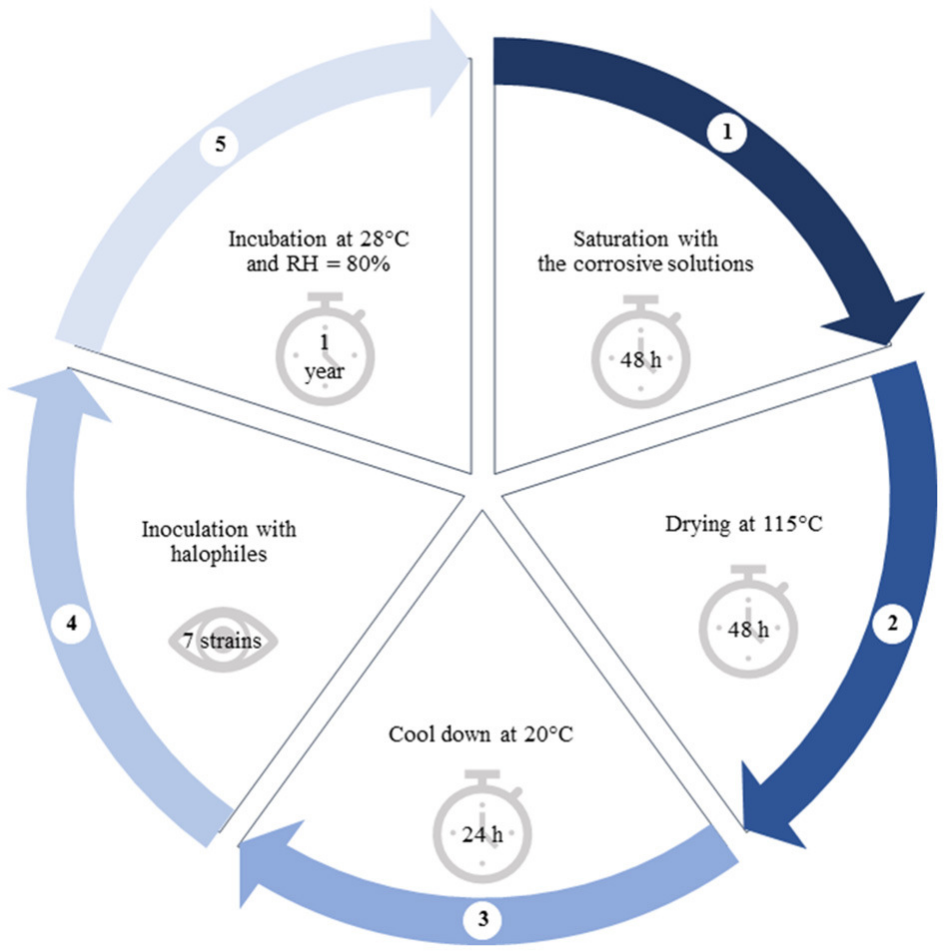
Diagram showing the following steps of brick sample preparation.

Samples (*n* = 9) of dimensions 65 × 65 × 65 mm were cut out of burnt ceramic brick, which physical properties, chemical and phase composition showed in Supplementary Materials 1 were determined according to (Stryszewska, 2014). Samples in duplicates were exposed to salty corrosive environment periodically (Stryszewska, 2014). Over 2 days each sample was immersed up to half of its height in the corrosive solution, i.e., potassium chloride (sample I), sodium chloride (II), magnesium sulfate (III) and magnesium nitrate (IV) of 50 g/dm^3^. As a result of capillary action, the samples were entirely saturated with salty corrosive solutions. For the next 2 days the samples were dried at 115°C and then cooled down at 20°C. A single cycle of exposure to the corrosive environment took 5 days in total (Figure 1). In this paper the authors present results after the full 1 cycle of exposure to all corrosive salt solutions (samples I_1 ÷ IV_1) and the full 3 cycles of the exposure only to sodium chloride for comparison (sample II_3).

#### Inoculation with halophilic microorganisms

One of the two samples that were saturated with the same salt solution was inoculated with the mixture of 7 strains of halophilic microorganisms at regular intervals throughout a year (Figure 1, Table 1) (Inoculated samples: I_1, II_1, III_1, IV_1, II_3). They were isolated from deteriorated historic buildings with visible symptoms of dampness and salt efflorescence and characterized in our previous studies (Adamiak et al., 2016; Otlewska et al., 2017). The second sample was served as a control (Uninoculated samples: I, II, III, IV). It was kept in the same conditions; however it was not inoculated with mixed cultures of halophilic microorganisms (samples I ÷ IV). The bacterial cultures were activated on TSB medium with both 10 % NaCl (w/v) and 2% MgSO_4_ × 7 H_2_O (w/v) and incubated aerobically at 30°C for 5 days. The cultures were centrifuged (6,000 × g, 20 min), and the biomass was suspended in M0 medium [(NH_4_)_2_SO_4_ 0.075%, K_2_HPO_4_ 0.025%, MgSO_4_× 7 H_2_O 0.125%, yeast extract 0.125%, glucose 0.5%, pH 6.0]. To obtain mixed cultures, bacterial strains were combined in equal volumes. The density of the suspension was adjusted to 10^8^ CFU/ml. The suspension was applied on the surface of brick samples. In order to promote growth and to elucidate biodeterioration caused by halophilic microorganisms, all samples were stored at 28°C and exposed to high humidity (RH = 80%) for a year. Untreated brick—unsalted and uninoculated, (The Fired Brick Production “Konstantynów” in Sanniki, Poland) was served as a reference sample (Sample K).

**Table 1:** The list of preselected halophilic microorganisms combined in order to inoculate the brick samples.

**Strains**	**GenBank accession number**	**Isolation area**	Inhabited historic buildings^*^	**References**
*Halobacillus styriensis*	KU550580.1	Brick^a^	Castles	Piñar et al., 2001; Ripka et al., 2006
*Halobacillus naozhouensis*	KU550581.1		Castles	Piñar et al., 2001; Ripka et al., 2006
*Staphylococcus succinus*	KU550582.1	Brick^b^	Wall paintings catacombs	Ettenauer et al., 2014; Piñar et al., 2014
*Halobacillus hunanensis*	KU550583.1		Catacombs	Piñar et al., 2014
*Marinococcus halophilus*	KU550584.1	Brick^c^	Wall paintings	Ettenauer et al., 2014
*Sterigmatomyces halophilus*	KU550579.1		nd	–
*Virgibacillus halodenitrificans*	KU550585.1	Plaster with paint coatings^d^	Catacombs walls of well	Xiang et al., 2008; Piñar et al., 2014

a ,Samples collected from historical buildings located in the former Auschwitz II-Birkenau concentration and extermination camp;

b,c,d *,samples collected from 19th century chateau in Łódz*.

* *Known from literature; nd, not detected in previous studies; – lack of data*.

### Metabolomic protocol

#### Extraction procedures

The field sample of 0.5 g of ground brick, was acidified to pH 2 with 3.0 ml of 4 N HCl (ACS grade, EMD Millipore) and sonicated in a water bath for 30 min. The mixtures were extracted into 3 ml ethyl acetate (HPLC grade, Sigma Aldrich), 2 times each and centrifuged at 1 × g for 5 min. The derived extracts were evaporated to dryness under ultrapure nitrogen gas (Grade 5.0) and dissolved in 100 μl of isopropanol (HPLC grade, Sigma Aldrich). The injection volume was 5 μl. Three biological replicates of each sample were analyzed (biological replicate in this study refers to an individual of the same group in the experiment), with *p*-value of 0.01 for abundance comparisons between the sets of triplicates.

#### HPLC/HRMS analysis

The metabolite extracts were analyzed on an Agilent 1290 Infinity HPLC coupled to an Agilent 6545 Ultra High Definition Q-TOF mass spectrometer. Samples were measured in both a positive and a negative ion mode. A SeQuant® ZIC®-HILIC column (particle diameter 5 μm, 150 × 4.6 mm, The Nest Group, Inc., Mass., USA) was used for LC separation of metabolites for HPLC/HRMS analysis in a positive ion mode with a flow rate 0.3 ml/min. The mobile phase was composed of A = LCMS grade water (EMD Millipore) with 0.1% formic acid and B = LCMS grade acetonitrile (EMD Millipore) with 0.1% formic acid (HPLC grade, Sigma Aldrich). A linear gradient elution from 80 to 20% acetonitrile was applied for 30 min, followed by 5% acetonitrile for the next 8 min. MS parameters were as follows: ion-source gas temperature 350°C, capillary voltage 3,500 V, fragmentor voltage 160 V, m/z range 50–1,100, data acquisition rate 4 GHz, 1 spectrum recorded per second. For separation in a negative ion mode, an Acquity UPLC® BEH C18 SB column (particle diameter 1.8 μm, 100 × 2.1 mm, Waters, Ireland) was used with a flow rate 0.4 ml/min. For the first 35 min, a linear gradient from 23.5 to 95.5% acetonitrile (LCMS grade, EMD Millipore) was used, followed by 5 min at 95.5%.

#### Data analysis

Raw data was analyzed using IDEOM v. 19 workflow (Creek et al., 2012) which enables processing of metabolomics data to annotated and hyperlinked metabolite lists. LC-MS files were processed in the R environment (https://www.r-project.org) (R, 2008), using XCMS centWave (Smith et al., 2006; Tautenhahn et al., 2008) and mzmatch.R tools (http://mzmatch.sourceforge.net/index.php) (Scheltema et al., 2011). Raw peaks were extracted by XCMS Centwave, while mzmatch.R allowed peak matching, noise filtering, gap-filling, annotation of related peaks and storage of the data in peakML files. Parameter settings were as follows: centWave for feature detection, Δ m/z = 10 ppm; minimum peak width = 15 s and maximum peak width = 100 s and parameters for chromatogram alignment, including a retention time window of 0.5 min and mass error window of 5 ppm. The result of the peak alignment is a list of compounds with associated mass and retention time.

Metabolite identification was achieved by matching the mass and retention time of observed peaks to metabolites in the database. i.e., KEGG, Kyoto Encyclopedia of Gens and Genomes, (Kanehisa et al., 2014), Metacyc (Caspi et al., 2014), Lipidmaps (Fahy et al., 2009) and HMDB (Wishart et al., 2013), with mass tolerance of 4 ppm. Retention times for authentic standards, and a retention time prediction model, were included for ZIC-HILIC chromatography data (Creek et al., 2011). The retention time calculator uses physic—chemical properties to predict retention times based on a multiple linear regression model with 120 authentic standards. (QSPR approach). To calibrate the retention time predictor 33 metabolite standards were used (Supplementary Materials 2). In our study, metabolites matching 5 of the standards were observed (written in bold; Supplementary Materials 2). The maximum difference between calculated and observed RT for authentic standards was 5%, whereas for other metabolites it was set to 45% (when experimentally observed retention time was more than 45% off from that calculated by the model, putative metabolites were rejected). With the use of retention time calculator, an identification level of 2 can be claimed (putatively annotated compounds; Sumner et al., 2007).

Multi group analysis, interpretation and visualization of statistically significant results was performed with MetaboAnalyst 3.0 (http://www.metaboanalyst.ca) and XCMS Online (http://xcmsonline.scripps.edu), which enabled the evaluation of the metabolite variation across different experimental groups and provided the univariate analysis of variance (one-way ANOVA) as a parametric test option. To display the metabolites identified in the context of metabolic pathways in which they occur, Pathos (http://motif.gla.ac.uk/Pathos/) and KEGG (http://www.genome.jp/kegg/pathway.html) were utilized.

#### β-carotene identification

β-carotene was analyzed with ultra-high-performance liquid chromatography-diode array detector-tandem mass spectrometry (UHPLC-DAD-ESI-MS). Chromatographic analyses were performed using UHPLC+ Dionex UltiMate 3000 system (Thermo Fisher Scientific Inc., Waltham, Massachusetts, USA), coupled to both a diode array detector with multiple-wavelength (Thermo Fisher Scientific Inc., USA), and Q-Exactive Orbitrap™ mass spectrometer (Thermo Scientific, USA). Accucore C30 column (particle diameter 2.6 μm, 100 × 3.0 mm, Thermo Fisher Scientific Inc., USA) was used. The column temperature was maintained at 35°C. The gradient elution was carried out using methanol (HPLC grade, J. T. Baker Chemicals) as solvent system A, methyl tert-butyl ether (HPLC grade, J. T. Baker Chemicals) as solvent system B and ultrapure water (EMD Millipore) as solvent system C, and applied as follows: 2% B and 2% C at 0-1 min, 80% B and 2% C at 35 min, 80-2% B and 2% C, over 35.0–38.0 min, and held at 2% B and 2% C until 50.0 min. The injection volume was 5 μL and the flow rate used was 0.4 ml/min. The chromatograms were recorded at processed at 450 nm. The mass spectrometer conditions were as follows: capillary temperature 350°C, heater gas temperature 250°C, electrospray capillary voltage 3.5 kV. The nebulizer gas and collision gas was nitrogen. The collision energy was 25 eV. Full-scan MS and target MS2 spectra were obtained by scanning m/z from 100 to 1,000. Instrument control, data acquisition and evaluation were done with the Qexactive Tune 2.1, Chromeleon 6.8 Chromatography Data System, and Thermo Xcalibur 2.2 software, respectively. Identification of β-carotene was performed by comparison of retention times UV-visible absorbance spectra characteristics, full scan mass spectra, and MS2 fragmentation patterns with those of the standards and the literature data (Mariutti et al., 2012).

### CARS microscopy

Inoculated brick samples, saturated with different salt solutions (samples I_1, II_1, II_3, III_1 and IV_1, respectively) were grounded and attach to Nunc™ Lab-Tek™ chamber slide (Thermo Fisher Scientific, USA). Microscopy analysis of each sample was based on the Coherent Anti-stokes Raman Scattering (CARS) technology, which involves two photon excitation to visualize the vibrational contrast of molecules in specimens. The multimodal non-linear microscope consisted of inverted microscope Leica DMi8 equipped with the Leica TCS SP8 CARS module and confocal module Leica SP8 (Leica Microsystems, Germany). The Leica TCS SP8 CARS uses a tunable pump laser with a tuning range from 780 to 940 nm, combined with a Stokes laser at 1064 nm, provided by Laser Pico Emerald integrated with Optical Parametric Oscillator (OPO) of 750 mW power. System covers all Raman shifts in the range of 1,250–3,400 cm^−1^ wavenumbers, divided into two filter set ranging from 1,250 to 2,000 cm^−1^ and 2,000–3,500 cm^−1^. Images were taken on a CARS filter set 2,000–3,500 cm^−1^ and analyzed with Leica software Leica Application Suite X (LAS X) Version 2.0. Settings were set as follows: (1) sequential scanning: scan1—bright field, scan 2—CARS; (2) objective: 63 ×; (3) immersion: water; (4) bright field scan on confocal microscope: white light laser, Ex. 488 nm, intensity: 15,0 / 20,0%, TLD (transmitted light detector); (5) CARS scan: Raman shift 2850 cm^−1^ optimal for lipid fraction in the sample, best signal for organic material probing the C-H vibration, PMT (photomultiplier tubes) detector. Zoom was adjusted individually to each sample due to different particle size.

### Optical measurements

Color parameters were checked for each inoculated sample, saturated with salt solutions (samples I_1, II_1, III_1, IV_1). Uninoculated samples immersed in corrosive salt solutions (samples I, II, III, IV) were served as controls. The measurements of color were performed with Chroma meter Minolta CR-400 with Spectra Magic NX 1.3 software (Konica Minolta, Japan) under the CIEL^*^a^*^b^*^ color space. The CIE (Commission International de l'Eclairage) color values, L^*^ (lightness, from 0-black to 100-white), a^*^ (from (−50)-green to 50-red) and b^*^ (from (−50)-blue to 50-yellow), were determined. The total color change (Δ*E*) was calculated as follows:

$$\begin{array}{l} \Delta E=\;\sqrt{{\Delta L}^{*\;2}+\;{\Delta a}^{*\;2}+\;{\Delta b}^{*\;2}} \end{array}$$

## Results and discussion

The main objective of the present study was to identify the pattern of metabolic responses by halophilic microorganisms exposed to different salts present in brick samples. For this purpose, we sought to isolate groups of metabolites potentially responsible for the degradation of building materials, as well as those that play a vital role in haloadaptation to severe saline conditions.

**Figure 2:**
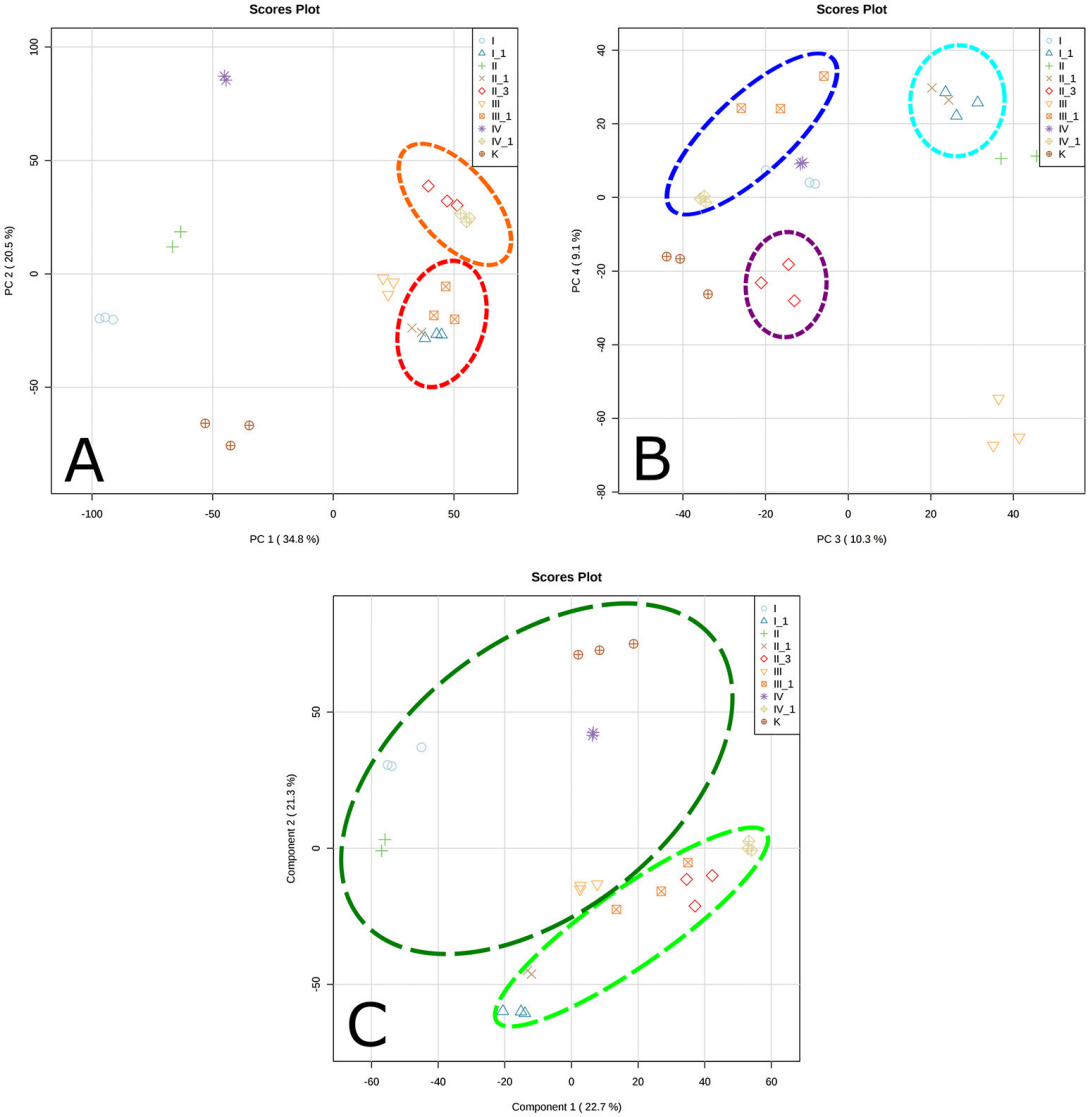
Principal Component Analysis (PCA) plot based on putatively identified metabolites (metabolomic data from each triplicate analysis of each of the samples): **(A)** PC 1 and PC 2, **(B)** PC 3 and PC 4, **(C)** corresponding PLS-DA plot; — cluster dominated by the inoculated brick samples exposed to corrosive effect of KCl, NaCl (1st cycle) and MgSO_4_; — cluster containing the inoculated samples saturated in Mg(NO_3_)_2_ and NaCl (3rd cycle); — cluster dominated by the inoculated NaCl and KCl samples; — cluster dominated by the inoculated MgSO_4_ and Mg(NO_3_)_2_ samples; — cluster containing the inoculated samples saturated in NaCl (3rd cycle); — cluster containing the inoculated samples; — cluster containing the uninoculated samples. I, KCl/uninoculated; I_1, KCl/inoculated; II, NaCl/uninoculated; II_1, NaCl (1 cycle of saturation)/inoculated; II_3, NaCl (3 cycles of saturation)/inoculated; III, MgSO_4_/uninoculated; III_1, MgSO_4_/inoculated; IV, Mg(NO_3_)_2_/uninoculated; IV_1, Mg(NO_3_)_2_/inoculated; K, unsaturated/uninoculated.

The metabolome was found to be largest in the sample saturated with MgSO_4_ (838 compounds) and smallest in the sample saturated with KCl solution (574 compounds). The numbers of putative identifications were noticeably different between samples (Table 2). Similarities and differences between the samples from different backgrounds separated into distinct clusters, as defined by principal component analysis (PCA) plots (Figure 2). According to the PCA model, total variance of the data was 55.3%, with 34.8% and 20.5% attributed to PC1 and PC2, respectively, and an additional 19.4% contributed by PC3 (10.3%) and PC4 (9.1%). PC1 and PC2 results (Figure 2A) show clear clustering of inoculated and uninoculated samples. The samples saturated with different salt solutions and inoculated with mixed cultures of halophilic microorganisms are spread across two different clusters. The first cluster (red dashed line) is dominated by the inoculated brick samples exposed to the corrosive effect of KCl, NaCl (1st cycle), and MgSO_4_. The second cluster (orange dashed line) contains exclusively the inoculated samples saturated with Mg(NO_3_)_2_ and NaCl (3rd cycle). By comparison, results defined by PC3 and PC4 (Figure 2B) show structured responses to different salt types. Chloride salts (blue dashed line) and magnesium salts (navy dashed line) are clustered separately, as are samples exposed to 3 cycles of NaCl (purple dashed line), which also form a distinct group. Such clustering may be explained by previous findings, whereby halophiles isolated from historical artifacts showed higher tolerance to magnesium salts (Oren, 1994; Saiz-Jimenez and Laiz, 2000). This feature may play an important role in haloadaptation (De Medicis, 1986). In contrast, a minimum concentration of Na^+^ ions is sufficient for growth, due to their impact on transport processes in the cell membrane (Ventosa et al., 1998). According to the literature, Mg^2+^ ions are required for activation of biosynthetic systems, whereas KCl and NaCl have a mostly inhibitory effect (Stern and Tietz, 1978; Oren, 2002). Here, PCA plots were constructed based on an unsupervised method and thus only a qualitative separation is showed. PCA allowed us to detect sample patterns and the differences between them. As the latter remained somewhat unclear, we used partial least squares discriminant analysis (PLS-DA) to achieve a better separation between samples and evaluate the causative factors for the observed clustering (Figure 2C). The PLS-DA plot based on putatively identified metabolites, revealed a clear separation between uninoculated (dark green dashed line) and inoculated samples (light green dashed line). Accordingly, the abundances (concentrations) of putative metabolites may be related to the microorganisms themselves and their ability to produce metabolites potentially associated with brick deterioration, rather than a specific type of salt. Figure 2 presents strong evidence for the existence of clear metabolic differentiation as a consequence of halophile response to a saline environment.

**Table 2:** Putative compounds in each sample.

**Sample**		**Total metabolome^a^**	**Putative identifications^b^**	**Discriminative metabolites^c^**
KCl		574	116	71
NaCl	1st cycle	698	119	36
	3rd cycle	685	117	148
MgSO_4_		838	171	161
Mg(NO_3_)_2_		810	173	113

a *The total number of compounds summed from each sample*.

b *In the total metabolome*.

c *In the total metabolome; metabolites involved in separation of inoculated sample cluster*.

Metabolomic data indicated the presence of intermediates presumably involved in 26 metabolic pathways related to both degradation of building materials and adaptation to severe saline environment (Table 3). The identified pathways included: biosynthesis and degradation of amino acids (e.g., valine, lysine, isoleucine), sulfur metabolism and carbohydrate digestion, carotenoid biosynthesis (salinixanthin), mineral absorption via sodium and chloride transporters, and glycerophospholipids metabolism, which plays a crucial role in the formation of biological membranes. A complete list of pathways and the detected intermediates is presented in Supplementary Materials 3.

**Table 3:** The list of metabolic pathways; annotated metabolites were involved as intermediates.

**No**.	**Pathway^a^**	**Metabolites involved as intermediates^b^**	**Formula**	**High abundance in samples^c^**
1	Nicotinate and nicotinamide metabolism	NAD+	C_21_H_28_N_7_O_14_P_2_	All
		Nicotinurate	C_8_H_8_N_2_O_3_	
2	Pantothenate and CoA biosynthesis	(R)-4′-Phosphopantothenoyl-L-cysteine	C_12_H_23_N_2_O_9_PS	KCl, NaCl, Mg(NO_3_)_2_
		L-Valine	C_5_H_11_NO_2_	
3	Aminoacyl-tRNA biosynthesis	L-Valine	C_5_H_11_NO_2_	KCl, NaCl, Mg(NO_3_)_2_
4	Carotenoid biosynthesis	Salinixanthin	C_51_H_78_O_8_	All
5	Mineral absorption via sodium- and chloride-dependent transporter	L-Valine	C_5_H_11_NO_2_	All
6	Cysteine and methionine metabolism	3-(Methylthio)propionic acid	C_4_H_8_O_2_S	Mg(NO_3_)_2_
7	Lysine biosynthesis	N-Succinyl-LL-2,6-diaminoheptanedioate	C_11_H_18_N_2_O_7_	NaCl, Mg(NO_3_)_2_
8	Lysine degradation	5-Aminopentanoate	C_5_H_11_NO_2_	KCl, NaCl, Mg(NO_3_)_2_
9	Oxidative phosphorylation	NAD+	C_21_H_28_N_7_O_14_P_2_	All
10	Photosynthesis	Plastoquinol-1	C_13_H_18_O_2_	NaCl
11	Sulfur metabolism	3-(Methylthio)propionic acid	C_4_H_8_O_2_S	Mg(NO_3_)_2_
12	Arginine and proline metabolism	5-Aminopentanoate	C_5_H_11_NO_2_	KCl, NaCl, Mg(NO_3_)_2_
13	Phenylalanine metabolism	Phenethylamine	C_8_H_11_N	KCl, NaCl, Mg(NO_3_)_2_
14	Thiamine metabolism	NAD+	C_21_H_28_N_7_O_14_P_2_	All
15	Valine, leucine and isoleucine biosynthesis	L-Valine	C_5_H_11_NO_2_	KCl, NaCl, Mg(NO_3_)_2_
16	Valine, leucine and isoleucine degradation	L-Valine	C_5_H_11_NO_2_	KCl, NaCl, Mg(NO_3_)_2_
17	Glycine, serine and threonine metabolism	Betaine	C_5_H_11_NO_2_	KCl, NaCl, MgSO_4_
18	Histidine metabolism	Ergothioneine	C_9_H_16_N_3_O_2_S	All
19	Glucosinolate biosynthesis	L-Valine	C_5_H_11_NO_2_	KCl, NaCl, Mg(NO_3_)_2_
20	Cyanoamino acid metabolism	L-Valine	C_5_H_11_NO_2_	KCl, NaCl, Mg(NO_3_)_2_
21	Butanoate metabolism	Butanoic acid	C_4_H_8_O_2_	KCl, NaCl, MgSO_4_, Mg(NO_3_)_2_
22	Methane metabolism	Methanofuran	C_34_H_44_N_4_O_15_	MgSO_4_
23	Sphingolipid metabolism	3-Dehydrosphinganine	C_18_H_37_NO_2_	KCl, MgSO_4_
		Phytosphingosine	C_18_H_39_NO_3_	
24	Glycerophospholipid metabolism	Phosphatidylcholine	C_10_H_18_NO_8_PR_2_	All
		Phosphatidylethanolamine	C_7_H_12_NO_8_PR_2_	
25	Primary bile acid biosynthesis	7alpha-Hydroxycholest-4-en-3-one	C_27_H_44_O_2_	All
		Chenodeoxycholate	C_24_H_40_O_4_	
26	Secondary bile acid biosynthesis	Chenodeoxycholate	C_24_H_40_O_4_	All

a *Metabolic pathways related to both degradation of building materials and adaptation to severe saline environment*.

b *Putatively annotated compounds, Level 2 (Sumner et al., 2007)*.

c *Inoculated brick samples saturated in the following corrosive salt solution*.

Metabolomics studies have provided answers to numerous basic questions regarding the adaptation of halophiles to salty environments and their ability to degrade building materials. In our study, we found metabolites potentially involved as intermediates in metabolic pathways such as synthesis of compatible solutes (betaine). This finding supports the mechanism allowing for osmoadaptation of halophilic microorganisms found in building materials. According to the “salt-in-cytoplasm” strategy, microorganisms adapt the protein machinery of the cell to augment the salt concentration in the cytoplasm. In contrast, identification of betaine synthesis pathways supports the action of the organic-osmolyte strategy, which is based on the uptake or synthesis of organic molecules so that their concentration within the cell is regulated according to the salt concentration outside the cell. The ability of aerobic heterotrophic Bacteria to synthesize betaine has not been proved yet; however, betaine synthesis was reported in phototrophic Bacteria and Archaea via the serine pathway with choline as an intermediate (Kunte et al., 2002). Besides playing a crucial role in haloadaptation, compatible solutes can stabilize proteins or even whole cells, protecting them against heat, desiccation, freezing and thawing, and denaturants such as urea (Lippert and Galinski, 1992; Kunte et al., 2002). Interestingly, it was found that members of the genus *Halobacillus*, which are mainly chloride-dependent bacteria, were able to switch their osmolyte strategy as a consequence of changing salinity (Ma et al., 2010). Moreover, our results showed also the biosynthesis of a ketocarotenoid similar to salinixanthin. One of the possible ways for its formation is synthesis from β-carotene. The formation of ketolated carotenoids from β-carotene is rather rare and was observed for the first time for marine bacteria (Ralley et al., 2004). UPLC coupled to diode-array detector electrospray ionization mass spectrometry (UHPLC-DAD-ESI-MS) of inoculated brick samples confirmed, however, the presence of β-carotene (Supplementary Materials 4), thus supporting the above-mentioned theory. β-carotene was present in every inoculated brick sample, but was most abundant in the sample saturated with KCl solution. This finding emphasizes the destructive potential of halophilic microorganisms, in particular their ability to cause aesthetic changes on the surface of buildings. The changes in color of inoculated brick samples immersed in corrosive salt solutions, were corroborated by spectrophotometric measurements using the CIEL^*^a^*^b^*^ system (Table 4). Thus, inoculation with halophilic microorganisms and yearlong incubation at 28°C and 80% relative humidity resulted in a statistically significant increase in brightness (L^*^), a decrease in redness (a^*^) and yellowness (b^*^), as well as a total color change exceeding a value of 5 on the surface of samples. Generally speaking, salty deposits mimic extreme saline habitats, hostile to halophilic microorganisms and contribute to further contamination of building materials, which may be observed as yellowish to reddish discolorations caused by production of carotenoids. Primarily, pigment formation is associated with a response against photooxidative damage, chemical, and/or salt stress. On salt-attacked objects, biogenic pigments are usually very resistant, even after the microorganisms' death (Piñar et al., 2014). Previous studies held by Ettenauer et al. (2014) and Piñar et al. (2014) on objects of art, revealed that microbial metabolites, especially pigments, altered building elements, and were thus major contributors to biodeterioration. Here, the biocorrosive potential of halophilic microorganisms was also proved by the formation of trace amounts of acids, in particular acetic acid (Supplementary Materials 5). Organic acids are known to be major causes of biodeterioration of building materials (Gutarowska and Czyzowska, 2009).

**Table 4:** Color parameters of brick samples.

**Corrosive salt solution**		**Uninoculated sample**			**Inoculated sample**		
		L^*^	a^*^	b^*^	L^*^	a^*^	b^*^
KCl		M: 43.35 *SD*: 0.89	M: 24.11 *SD*: 0.64	M: 25.51 *SD*: 0.65	M^a^: 44.64 *SD*: 1.04	M^a^: 19.38 *SD*: 0.92	M^a^: 19.94 *SD*: 0.74
					ΔE: 7.43		
NaCl	1st cycle				M^a^: 45.36	M^a^: 19.91	M^a^: 19.81
					*SD*: 0.80	*SD*: 1.29	*SD*: 1.07
		M: 43.35	M: 24.11	M: 25.51	ΔE: 7.36		
	3rd cycle	*SD*: 0.89	*SD*: 0.64	*SD*: 0.65	M^a^: 36.75 *SD*: 1.65	M^a^: 19.96 *SD*: 1.11	M^a^: 20.97 *SD*: 1.26
					ΔE: 9.02		
MgSO_4_		M: 43.29 *SD*: 1.63	M: 23.16 *SD*: 0.74	M: 23.64 *SD*: 0.98	M^a^: 48.59 *SD*: 0.79	M^a^: 17.67 *SD*: 0.97	M^a^: 18.50 *SD*: 0.31
					ΔE: 9.20		
Mg(NO_3_)_2_		M: 41.85 *SD*: 0.64	M: 20.73 *SD*: 1.36	M: 21.98 *SD*: 0.75	M^a^: 45.38 *SD*: 1.49	M^a^: 15.24 *SD*: 1.14	M^a^: 18.52 *SD*: 1.30
					ΔE: 7.39		

M, arithmetic mean; SD, standard deviation; L^*^, lightness; a^*^, red-green axis; b^*^, yellow-blue axis; ΔE, total color change.

a *Statistically significant differences between inoculated samples saturated in corrosive salt solutions and uninoculated samples saturated in corrosive salt solutions (analysis of variance, ANOVA, p < 0.05)*.

**Figure 3:**
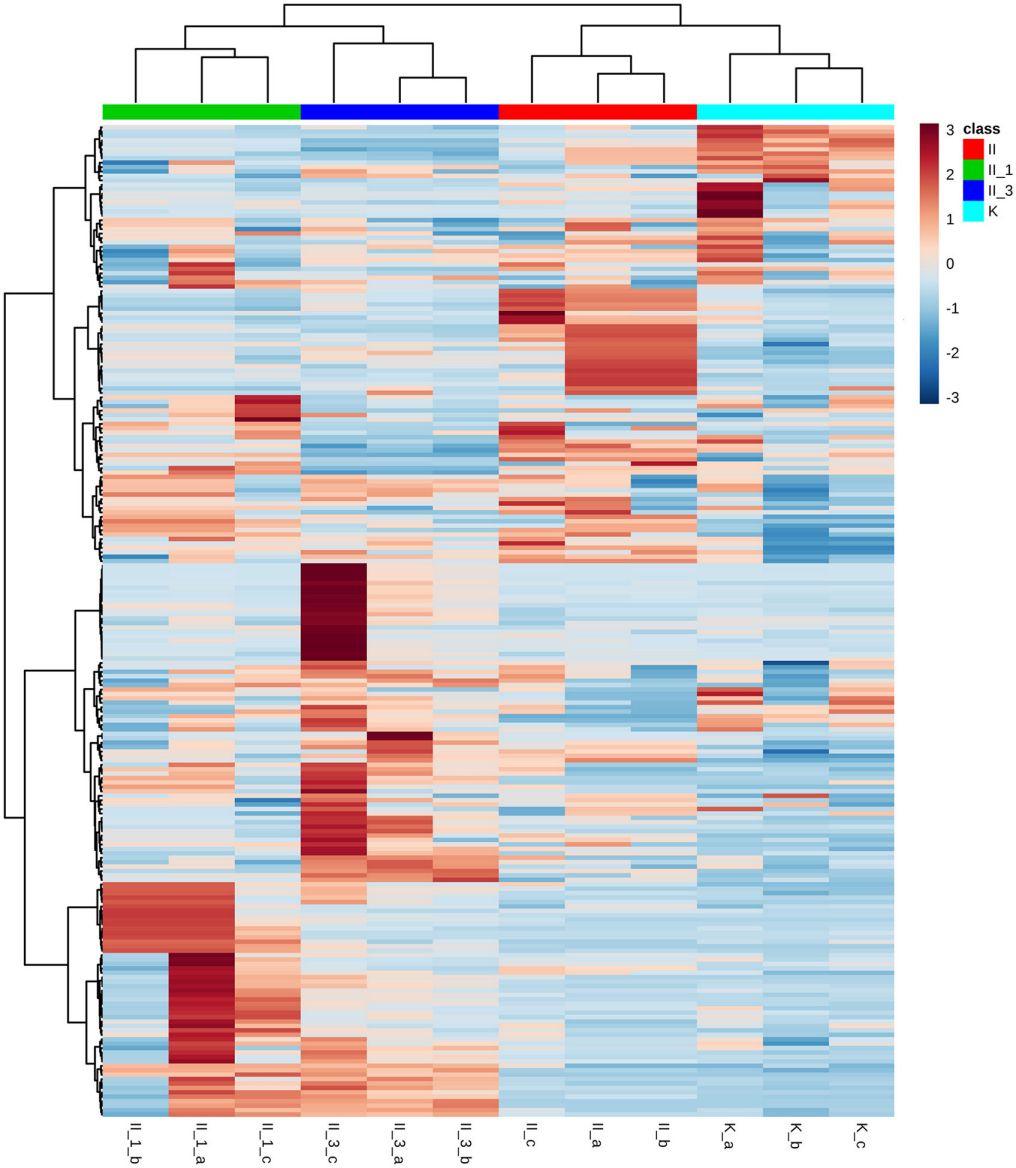
Hierarchical clustering with lipid structure visualization; distance measure: Euclidean; clustering algorithm: Ward. Each row represents a feature, while each column represents a sample. The blue color of the tile indicates low abundance and red indicates high abundance. II, NaCl/uninoculated; II_1, NaCl (1 cycle of saturation)/inoculated; II_3, NaCl (3 cycles of saturation)/inoculated; K, unsaturated/uninoculated; letters a,b,c refer to the following biological replicates.

Among putative metabolites, lipids were one of the dominant compound groups. They provide information pertaining to an organism's actual phenotype, variety of structural and functional properties, cellular architecture, and function (Harkewicz and Dennis, 2011). Among lipids, phospholipids (PLs) were the most abundant category, particularly neutrally charged phosphatidylethanolamine (PE) and phosphatidylcholine (PC), and negatively charged phosphatidylserine (PS), phosphatidylinositol (PI), and phosphatidylglycerol (PG). As expected, hierarchical clustering (Figure 3) showed a different lipid pattern in the sample exposed to higher salinity (sample II_3). Exposure to 3 cycles of a NaCl corrosive environment induced a decrease in the concentration of PS, while only slightly reducing the concentration of PE. A specific PL composition may be essential for haloadaptation (Oren, 2002). Any change in lipid composition resulting from exposure to a saline environment is due to preservation of the membrane's bilayer structure. To preserve its integrity, a correct proportion of bilayer-forming and non-bilayer-forming PLs is required. Generally, PE tends to form non-bilayer phases, whereas PG forms a stable bilayer. In this respect, increasing salinity results in an increase of negatively charged PLs at the expense of PE. Accordingly, the slightly lower PE concentration observed in our study when salinity was augmented, fits in part with this pattern, but does not allow us to draw any general conclusions. Interestingly enough, we noted a remarkable change in membrane composition due to a decrease in PS levels. This shift may be explained by immediate conversion to PE, of which PS is a precursor (Vance and Steenbergen, 2005). Furthermore, an increase in medium salinity induced an increase in the concentration of unsaturated fatty acids in the membrane. The major fatty acids detected in the sample with higher NaCl concentration (II_3) were C24:2^Δ5, 13^ and C20:1^Δ13^. Changes in fatty acid composition represent an important factor in the maintenance of membrane fluidity (Turk et al., 2004). Unsaturated fatty acids generally increase membrane fluidity (Oren, 2002).

**Figure 4:**
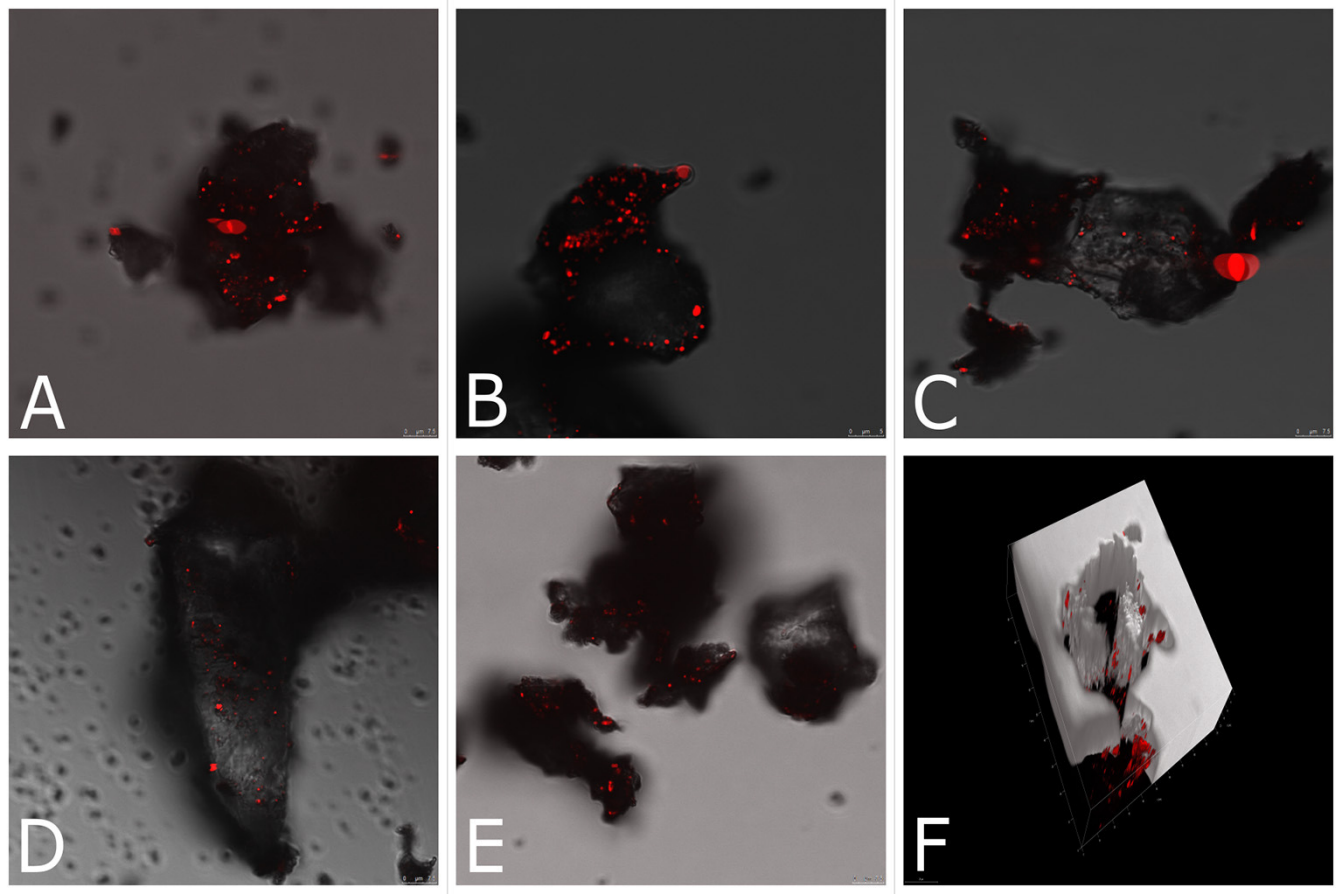
Lipid-based CARS imaging of inoculated brick samples saturated in the following corrosive salt solutions: **(A)** Mg(NO_3_)_2_, **(B)** MgSO_4_, **(C)** KCl, **(D)** NaCl 1st cycle of saturation, **(E)** NaCl 3rd cycle of saturation; the bright signals result from the CH_2_ symmetric stretch vibration of aliphatic lipid of the cells; **(F)** 3D distribution of halophiles across the KCl sample, dark interior depicts analyzed sample.

Finally, we applied CARS microscopy to elucidate biodeterioration and detect single halophilic microorganisms involved in this process *in situ*. Figures 4A–E shows a 2,850 cm^−1^ CARS image of the mixture of halophilic microorganisms in building material (red points in the picture). The corresponding 3D image (Figure 4F) provides a full picture of halophiles' distribution across the sample. Specifically, we examined the influence of environmental conditions (saturation in different salt solutions) on the abundance of halophilic microorganisms in building material. It is noteworthy, that in samples saturated with magnesium salts (Figures 4A,B) halophilic microorganisms are more abundant than in samples saturated with chloride salts (Figures 4C–E), which may be due to the aforementioned inhibitory effect of KCl and NaCl. The average diameter of the cell was 0.80 ± 0.08 μm. According to the 3D image (Figure 4F), halophiles were present across the entire sample (up to 25 μm). Although, the present study focused on halophiles detection, their metabolic activity may be studied by monitoring spectral changes (Hellerer et al., 2007) and thus provide clues of how haloadaptation occurs. In particular, viability of halophiles entrapped in small inclusions, following drying and the formation of crystals on the surface of building material, is a key topic in haloadaptation (Ma et al., 2010).

## Conclusions and future challenges

The main focus of the present study was to find a relationship between metabolomic data and activity of halophilic microorganisms in an attempt to better understand how they contribute to biodeterioration of building materials or cope with osmotic stress. Our results clearly demonstrate the usefulness of halophilic community metabolomics. The information produced by metabolomics and its very close link to the phenotype of microorganisms makes it possible to successfully find biomarkers, which could be used for early detection of biodeterioration. In the case of halophiles, carotenoids represent possible candidates. Although we found several metabolites, many of them remained unidentified. Therefore, a reliable evaluation of biodeterioration caused by halophilic microorganisms will necessarily have to extend beyond already identified metabolites. Of particular relevance for biodeterioration studies is to combine various metabolomics methods, separation modes, and platforms to significantly improve data integration on the role played by halophilic microorganisms. This may also reveal other mechanisms of action and complex interactions in a salty environment, as the one encountered in building materials. Until now, it was challenging to rapidly detect microbes in their natural environment; however, CARS microscopy offers unique advantages in this sense. Indeed, CARS may soon become an indispensable tool for lipid-related *in situ* detection of a single living cell with high sensitivity and selectivity, without alterations to the sample. In the future, it may become a crucial instrument for assessing biodeterioration of cultural property caused by halophilic microorganisms, where the amount of a sample is significantly limited.

## Author contributions

JA was responsible for MS analyses, data analysis, CARS microscopy analysis and took the lead in writing the manuscript. VB conducted MS analyses and assisted with data analysis and writing. AO took part in sample preparation, in particular preselection of strains and inoculation, assisted with CARS microscopy and manuscript proofreading. JS guided the MS analyses and contributed to the results proofreading. IB contributed to the MS analysis and results proofreading. TS was responsible for the brick sample preparation, especially saturation with salty corrosive solutions. SK was involved in collecting the brick samples and their further preparation. JO was involved in β-carotene identification, optical measurements and manuscript proofreading. DŻ contributed to the determination of color parameters and manuscript proofreading. BG contributed to the ideas, writing and editing the manuscript.

### Conflict of interest statement

The authors declare that the research was conducted in the absence of any commercial or financial relationships that could be construed as a potential conflict of interest. The reviewer TC and handling Editor declared their shared affiliation.
