# Supplementary material for: Untargeted Metabolomics Approach in Halophiles: Understanding the Biodeterioration Process of Building Materials
Source: Front Microbiol. 2017 Dec 11;8:2448. doi: 10.3389/fmicb.2017.02448 (PMC5732225; doi:10.3389/fmicb.2017.02448)
Supplement: Supplementary file 1 [file DataSheet1.DOCX]

Supplementary materials 1. Brick properties and composition.

| Physical properties | | Chemical composition [%] | | Phase composition [%] | |
| --- | --- | --- | --- | --- | --- |
| Bulk density  Skeleton density  Water absorption by weight  Total porosity  Open porosity  Close porosity | 1.8 g/cm^3^  2.7 g/cm^3^  14.3%  31.6%  26.2%  5.4% | SiO_2_  Al_2_O_3_  Fe_2_O_3_  CaO  MgO  Cr_2_O_3_  MnO  K_2_O  P_2_O_5_  SO_3_  Na_2_O  TiO_2_  ZrO_2_  Ignition losses | 64.8  16.0  5.7  2.6  3.5  <0.1  0.1  3.9  0.1  0.2  0.5  0.7  <0.1  1.5 | Quarz  Albite  Microcline  Calcite  Montmorrillonit | 59.0  7.0  16.0  2.4  7.0 |

Supplementary materials 2. List of authentic standard used for the calibration of retention time prediction model with ZIC-HILIC chromatography.

| Metabolites | RT |
| --- | --- |
| Benzoate | 5.68 |
| Fumarate | 6.21 |
| Pyruvate | 6.73 |
| Succinate | 6.73 |
| Uracil | 8.03 |
| L‐Rhamnose | 11.35 |
| L‐Phenylalanine | 11.84 |
| L‐Tryptophan | 12.26 |
| L‐Leucine | 12.63 |
| Guanosine | 12.86 |
| L‐Methionine | 13.11 |
| **L‐Proline** | 13.96 |
| **L‐Valine** | 14.02 |
| D‐Gluconic Acid | 14.52 |
| D‐Glucose | 14.97 |
| L‐Homocysteine | 15.97 |
| L‐Glutamate | 16 |
| L‐Tyrosine | 16.03 |
| L‐Cysteate | 16.08 |
| Taurine | 16.58 |
| L‐Threonine | 16.66 |
| **L‐Aspartate** | 16.69 |
| L‐Alanine | 18.51 |
| L‐Glutamine | 19.02 |
| Creatinine | 19.1 |
| **L‐Asparagine** | 19.47 |
| L‐Serine | 19.62 |
| Glycine | 19.62 |
| L‐Cystine | 23.22 |
| L‐Histidine | 26.25 |
| **L‐Arginine** | 27.29 |
| L‐Lysine | 27.67 |
| Thiamin | 33.43 |

Metabolites matching 5 of the standards were observed (written in bold).

Supplementary materials 3. Detection of metabolites involved in common metabolic pathways.

| Pathway^1^ | Metabolites detected/Metabolites in the pathway^2^ |
| --- | --- |
| Aminoacyl-tRNA biosynthesis | 2 / 24 |
| Anthocyanin biosynthesis | 1 / 56 |
| Arachidonic acid metabolism | 16 / 74 |
| Arginine and proline metabolism | 1 / 67 |
| Biosynthesis of 12-, 14- and 16-membered macrolides | 2 / 75 |
| Biosynthesis of ansamycins | 1 / 24 |
| Biosynthesis of siderophore group nonribosomal peptides | 1 / 13 |
| Biosynthesis of type II polyketide products | 1 / 86 |
| Brassinosteroid biosynthesis | 2 / 27 |
| Butanoate metabolism | 1 / 38 |
| Carotenoid biosynthesis | 1 / 91 |
| Caprolactam degradation | 1 / 24 |
| Cyanoamino acid metabolism | 1 / 24 |
| Cysteine and methionine metabolism | 1 / 54 |
| Diterpenoid biosynthesis | 4 / 69 |
| Glucosinolate biosynthesis | 2 / 72 |
| Glycerophospholipid metabolism | 2 / 57 |
| Glycine, serine and threonine metabolism | 1 / 45 |
| Histidine metabolism | 1 / 44 |
| Insect hormone biosynthesis | 1 / 23 |
| Limonene and pinene degradation | 5 / 59 |
| Lysine biosynthesis | 1 / 26 |
| Lysine degradation | 1 / 42 |
| Methane metabolism | 1 / 60 |
| Mineral absorption via sodium- and chloride- dependent transporter | 1 / 18 |
| Monoterpenoid biosynthesis | 3 / 44 |
| Naphthalene and anthracene degradation | 1 / 60 |
| Nicotinate and nicotinamide metabolism | 2 / 46 |
| Oxidative phosphorylation | 1 / 13 |
| Pantothenate and CoA biosynthesis | 2 / 25 |
| Penicillin and cephalosporin biosynthesis | 1 / 16 |
| Phenylalanine metabolism | 1 / 64 |
| Photosynthesis | 1 / 10 |
| Porphyrin and chlorophyll metabolism | 2 / 93 |
| Primary bile acid biosynthesis | 2 / 47 |
| Retinol metabolism | 4 / 17 |
| Secondary bile acid biosynthesis | 1 / 11 |
| Sesquiterpenoid biosynthesis | 4 / 34 |
| Sphingolipid metabolism | 2 / 11 |
| Steroid biosynthesis | 10 / 45 |
| Steroid hormone biosynthesis | 1 / 99 |
| Stilbenoid, diarylheptanoid and gingerol biosynthesis | 1 / 24 |
| Sulfur metabolism | 1 / 22 |
| Terpenoid backbone biosynthesis | 1 / 30 |
| Thiamine metabolism | 1 / 22 |
| Toluene and xylene degradation | 1 / 38 |
| Tropane, piperidine and pyridine alkaloid biosynthesis | 1 / 61 |
| Valine, leucine and isoleucine biosynthesis | 2 / 23 |
| Valine, leucine and isoleucine degradation | 2 / 32 |

^1^ Pathways are defined as in the KEGG database.

^2^ The number of metabolites detected by HPLC/HRMS and the total number of metabolites listed for that pathway.
