# Supplementary figures and images for: Untargeted Metabolomics Approach in Halophiles: Understanding the Biodeterioration Process of Building Materials
Source: Front Microbiol. 2017 Dec 11;8:2448. doi: 10.3389/fmicb.2017.02448 (PMC5732225; doi:10.3389/fmicb.2017.02448)

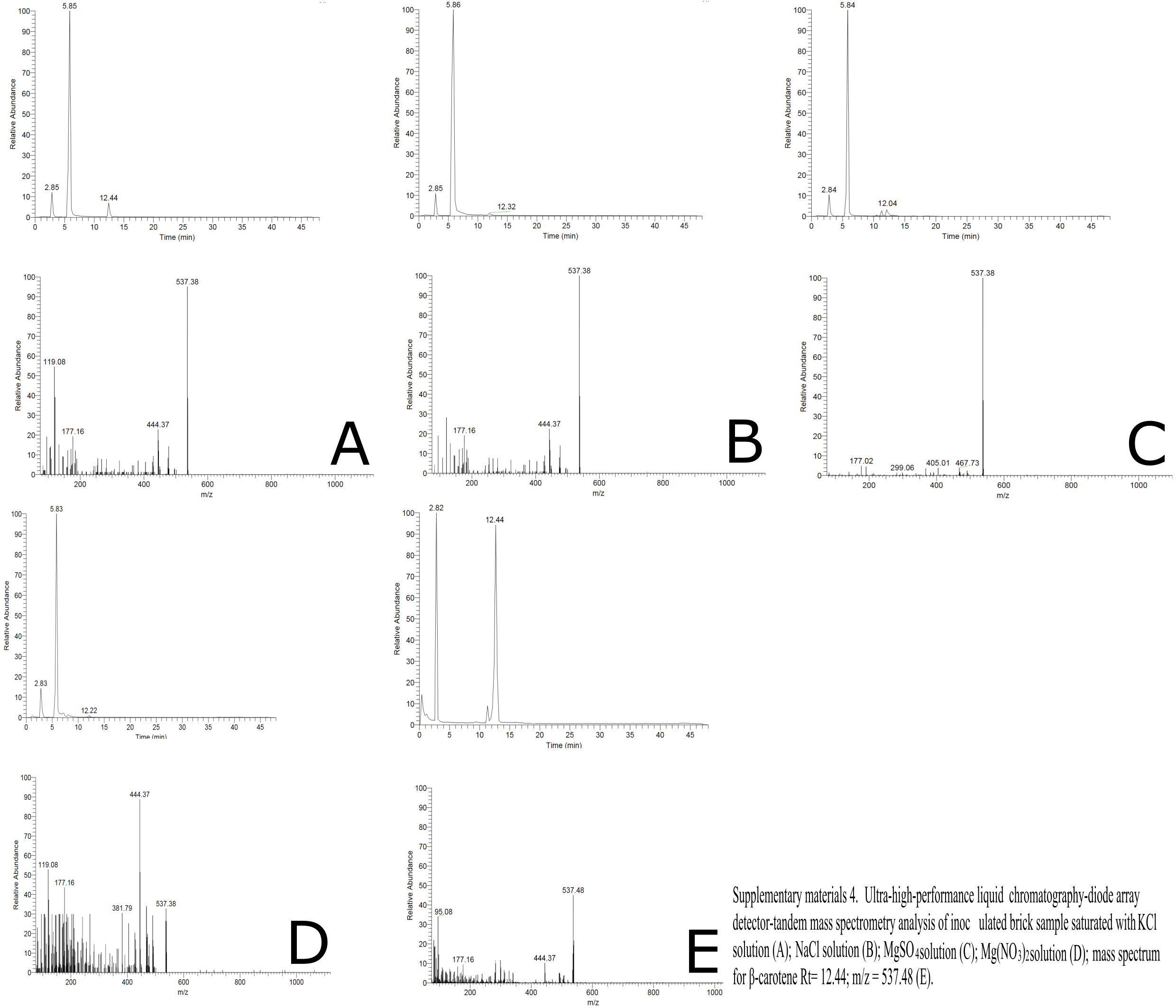

Supplement: Supplementary file 3 [file Image1.TIF]
